# Malignancy in Chronic Leg Wounds: Diagnostic Delay and Clinical Implications in a Tertiary Wound‐Care Cohort

**DOI:** 10.1111/iwj.70991

**Published:** 2026-07-02

**Authors:** Marcela Nowak, Dorota Piechota, Wioletta Barańska‐Rybak

**Affiliations:** ^1^ Department of Biomedical and Clinical Sciences Linköping University Linköping Sweden; ^2^ Clinical Department of Dermatology and Venereology in Östergötland Region Östergötland Linköping Sweden; ^3^ Department of Dermatology, Venereology and Allergology Medical University of Gdansk Gdansk Poland

**Keywords:** chronic leg wound, diagnostic delay, malignant wound, wound biopsy, wound oncology

## Abstract

Chronic leg wounds are common in wound‐care practice and are most often attributed to vascular or inflammatory etiologies. Rarely, they may represent or conceal underlying malignancy, and delayed recognition can lead to inappropriate management and poor outcomes. We aimed to evaluate diagnostic delay, clinical warning signs, histopathological findings, management strategies, and outcomes in patients with malignancy‐associated chronic leg wounds treated in a tertiary wound‐care setting. We conducted a retrospective observational study of consecutive patients with chronic lower‐leg wounds (> 6 weeks' duration) diagnosed with underlying malignancy between 2015 and 2022. Clinical characteristics, time to biopsy, histopathological findings, treatment, and outcomes were analysed descriptively. Among 328 patients with chronic leg wounds, eight (2.4%) were diagnosed with malignant etiologies. Mean ulcer duration at biopsy was 11.3 months (range 6–24), exceeding guideline‐recommended timelines. All wounds failed to heal despite standard care. Common red‐flag features included disproportionate pain, necrosis or bleeding, and atypical appearance. Earlier biopsy and multidisciplinary management were associated with improved wound outcomes, whereas prolonged diagnostic delay correlated with advanced disease and poor prognosis. Malignancy should be considered in any chronic leg wound failing to improve within 4–12 weeks or showing atypical features, and early biopsy is essential to optimise outcomes.

## Key Points

- Malignancy was found in 2.4% of all leg ulcers.
- The mean time to diagnostic biopsy was 11.3 months.
- Early biopsy may improve diagnosis and clinical outcomes.

## 1 Introduction

Chronic leg wounds represent a substantial clinical and economic burden, affecting up to 1%–2% of the adult population and disproportionately impacting older individuals with comorbid vascular disease [1]. The majority of cases are attributed to venous, arterial, or mixed aetiologies; however, a small but clinically significant proportion are associated with underlying malignancy [2, 3].

Malignant wounds may arise through malignant transformation of long‐standing wounds or scars—commonly referred to as Marjolin ulcer—or may represent primary cutaneous malignancies or cutaneous metastases that are initially misclassified as benign chronic wounds [4, 5, 6]. Differentiating benign from malignant chronic wounds is challenging, as both may present with delayed healing, exudation, necrosis, pain, and secondary infection. Consequently, malignancy is frequently excluded from the initial differential diagnosis, resulting in delayed biopsy and referral [2, 7].

Reported rates of malignancy among chronic leg wounds range from approximately 2% to 4% in selected cohorts, suggesting that while uncommon, this complication is clinically important [2, 8]. Current guidelines from the European Wound Management Association (EWMA) recommend biopsy of non‐healing wounds that fail to improve within 4–12 weeks of optimal therapy or exhibit atypical clinical features, such as unusual wound bed and edges (violaceous borders, undermined edges, vegetative granulation and stellate shape), pain out of proportion or atypical perilesional skin (satellite lesions, livedo reticularis, palpable purpura) [9]. Despite these recommendations, malignant wounds remain under‐recognised in routine wound‐care practice.

## 2 Study Aim

The aim of this study was to systematically evaluate diagnostic delay, clinical warning signs, histopathological diagnoses, management strategies, and outcomes in patients with malignancy‐associated chronic leg wounds. By analysing a consecutive cohort treated at a tertiary wound‐care center, we sought to identify features that may facilitate earlier recognition and improve interdisciplinary management.

## 3 Methods

We conducted a retrospective observational study at a tertiary university hospital, defined as a specialised referral center providing advanced care for complex and treatment‐refractory conditions, including chronic wounds. Hospital records were screened for patients coded under ICD‐10 code L97 (‘non‐pressure chronic ulcer of lower limb, not elsewhere classified’) between January 2015 and December 2022. Chronic wounds were defined as wounds persisting for more than 6 weeks [9].

Among 328 identified patients with chronic leg wounds, eight patients were ultimately diagnosed with an underlying malignant aetiology and met inclusion criteria. Patients with non‐malignant ulcer diagnoses were excluded.

Collected variables included age, sex, wound duration and location, clinical wound characteristics (pain, necrosis, bleeding, and border morphology, defined as the appearance and configuration of the wound edge, including raised, rolled, undermined, violaceous, or indurated borders), time from wound onset and from wound‐clinic presentation to diagnostic biopsy, histopathological and immunohistochemical findings (including malignant cell type, degree of atypia, invasion pattern, and expression of relevant immunohistochemical markers), treatment modalities, and clinical outcomes.

Primary outcomes were time to diagnostic biopsy and wound outcome, defined as complete wound healing, persistent nonhealing wound, or wound progression during follow‐up. Secondary outcomes included malignancy type, treatment strategy, and survival status at last follow‐up.

Ethical approval was obtained retrospectively (decision KB/643/2023). Informed consent was waived due to the retrospective and observational nature of the study.

## 4 Results

### 4.1 Cohort Characteristics and Diagnostic Patterns

Of 328 patients treated for chronic lower‐leg wounds during the study period, eight patients (2.4%) were diagnosed with an underlying malignant aetiology. The cohort comprised five men and three women, with a mean age of 71 years (range 48–82). All wounds were initially managed as benign chronic ulcers, most presumed venous ulcers.

The mean duration of wound at the time of diagnostic biopsy was 11.3 months (range 6–24). The mean interval from first presentation in the wound clinic to biopsy was 7.6 months (range 3–14). No patient underwent biopsy within 3 months of non‐healing.

Failure to respond to standard wound care, including regular wound cleansing, appropriate dressings, infection control, pressure offloading, compression therapy when indicated, and local topical treatments, was observed in all patients. Frequently documented red‐flag features included disproportionate or escalating pain (75% of patients), necrosis or spontaneous bleeding (63% of patients), and atypical clinical morphology such as violaceous plaques or nodular components (75% of patients).

**Detailed individual case descriptions are provided in** Table S1.

### 4.2 Malignancy Spectrum and Management Outcomes

Histopathological examination revealed a heterogeneous spectrum of malignancies. Epithelial malignancy accounted for one case of squamous cell carcinoma arising in a chronic scar (Marjolin ulcer). Non‐epithelial malignancies predominated and included hematologic malignancies (mycosis fungoides with ulceration, *n* = 3; primary cutaneous large B‐cell lymphoma, leg type, *n* = 1), vascular malignancy (angiosarcoma, *n* = 1), classic Kaposi sarcoma (*n* = 1), and metastatic carcinoma of unknown primary (*n* = 1).

Immunohistochemical analysis was essential for definitive diagnosis in all of those non‐epithelial cases. Treatment strategies varied according to malignancy type and disease stage but consistently required integration of oncologic therapy and specialised wound care.

### 4.3 Association Between Diagnostic Delay and Outcome

Prolonged diagnostic delay was associated with increased disease severity at presentation and limited therapeutic options. Wounds biopsied after more than 12 months of persistence were more frequently associated with aggressive malignancies or palliative management strategies, particularly angiosarcoma and metastatic carcinoma. Better outcome at patients with earlier biopsies need to be interpreted cautiously, as these were also patients with less aggressive underlying diagnosis.

## 5 Discussion

This study highlights the persistent diagnostic challenge posed by malignancy‐associated chronic leg wounds in routine wound‐care practice. Despite clear guideline recommendations advocating early biopsy of non‐healing wounds, substantial diagnostic delays were observed across the cohort, with all patients undergoing biopsy well beyond the recommended 4–12‐week timeframe [9]. No formal biopsy protocol was in place during the study period; the introduction of a structured biopsy decision checklist with defined review timepoints represents a priority area for quality improvement. These delays were associated with advanced disease at diagnosis and, in several cases, poor clinical outcomes.

Failure to heal despite appropriate standard care emerged as the most consistent warning sign of underlying malignancy, corroborating findings from previous studies [2, 4, 7]. Additional features—such as disproportionate pain, necrosis or bleeding, and atypical wound morphology—were frequently present and should prompt early histopathological evaluation. Importantly, the spectrum of malignancies encountered extended well beyond squamous cell carcinoma, underscoring the need for clinicians to maintain a broad differential diagnosis [4, 5, 6, 8, 9, 10, 11].

Several factors may contribute to delayed biopsy in chronic leg ulcers despite existing guideline recommendations. In routine clinical practice, chronic wounds are most commonly attributed to venous or arterial etiologies, which may lead to anchoring bias and prolonged continuation of conservative management in the absence of early suspicion for malignancy. In addition, overlapping clinical features between malignant and non‐malignant wounds, such as pain, exudation, and delayed healing, may further obscure early recognition. System‐level factors may also play a role, including delayed referral pathways, limited access to timely biopsy services, and variability in awareness of malignant transformation among non‐specialist clinicians managing chronic wounds. Finally, the progressive and indolent nature of some cutaneous malignancies may contribute to a false perception of stability during early disease stages, further delaying diagnostic intervention. While these factors were not directly assessed in the present study, they represent plausible contributors to the observed diagnostic delays and highlight areas for future prospective investigation.

Histopathological and immunohistochemical analyses were indispensable for establishing definitive diagnoses, particularly in non‐epithelial malignancies [12, 13]. These findings reinforce the importance of adequate biopsy technique, sampling from the wound edge, and repeat biopsy when clinical suspicion persists despite initial negative results [9, 12].

From a management perspective, our findings demonstrate that standard wound‐care interventions alone are insufficient in the presence of underlying malignancy. Early multidisciplinary collaboration between wound‐care specialists, dermatologists, oncologists, pathologists, and surgeons was critical to achieving disease control and optimising wound outcomes [6]. In advanced cases, a palliative approach focused on symptom relief and quality of life was required [14].

Limitations of this study include its retrospective design, small sample size and single‐center setting. Nevertheless, the consecutive nature of the cohort and detailed clinical characterization strengthen the relevance of the findings. Prospective multicenter studies are warranted to refine diagnostic algorithms and assess the impact of early biopsy strategies on outcomes.

## 6 Conclusions

Underlying malignancy should be considered in any chronic leg wounds that fails to demonstrate meaningful healing within 4–12 weeks or exhibits atypical clinical features. Early biopsy and prompt multidisciplinary management are essential to reduce diagnostic delay, guide appropriate therapy, and improve patient outcomes.

## Funding

The authors have nothing to report.

## Ethics Statement

Ethical committee has approved the study retroactively (decision KB/643/2023), stating that ‘the research does not raise any objections from the bioethics committee and does not require the opinion of the bioethics committee, as the study was purely observational and based on medical documentation’. The definitive diagnosis was pre‐existing, and the research was carried out from the documentation.

## Conflicts of Interest

The authors declare no conflicts of interest.

## Supporting information


**Table S1:** Detailed individual case descriptions of malignancy‐associated chronic leg ulcers.

## Data Availability

The data that supports the findings of this study are available in the Supporting Information of this article.
