# Supplementary material for: Malignancy in Chronic Leg Wounds: Diagnostic Delay and Clinical Implications in a Tertiary Wound‐Care Cohort
Source: Int Wound J. 2026 Jul 2;23(7):e70991. doi: 10.1111/iwj.70991 (PMC13325010; doi:10.1111/iwj.70991)
Supplement: Supplementary file 1 — Table S1: Detailed individual case descriptions of malignancy‐associated chronic leg ulcers. [file IWJ-23-e70991-s001.docx]

Supplementary Table S1. Detailed Individual Case Descriptions of Malignancy-Associated Chronic Leg Ulcers

| Case | Age / Sex | Ulcer duration at biopsy | Key clinical features | Initial presumed diagnosis | Histopathological diagnosis | Treatment | Outcome |
| --- | --- | --- | --- | --- | --- | --- | --- |
| 1 | 81 / F | 24 months | Rapidly enlarging ulcer, black necrotic crusts, bleeding, severe pain (VAS 7/10), marked oedema | Venous ulcer | Angiosarcoma (CD31+, ERG+, FLI-1+) | Debridement, hyperbaric oxygen (prior); oncology referral delayed | Disease progression; patient died before oncologic therapy |
| 2 | 48 / M | 9 months | Multiple chronic ulcerations within plaques, moderate pain (VAS 5/10), eosinophilia | Infection / eczema | Mycosis fungoides with ulceration (CD3+, CD4+, loss of CD7, TCR clonality) | Systemic bexarotene, topical therapy, wound care | Partial improvement with recurrent flares |
| 3 | 82 / F | 7 months | Purplish-red nodules progressing to ulceration, pain (VAS 6/10), elevated LDH | Inflammatory / vascular ulcer | Primary cutaneous large B-cell lymphoma, leg type (CD20+, BCL2+, MUM1+) | R-CHOP chemotherapy, wound care | Ulcer healing achieved; chemotherapy-related adverse effects |
| 4 | 80 / M | 6 months | Violaceous plaques with ulceration, mild pain (VAS 3/10), anaemia | Inflammatory ulcer | Classic Kaposi sarcoma (HHV-8+) | Intralesional vinblastine, compression therapy | Lesion regression; improved wound healing |
| 5 | 72 / M | 16 months | Persistent painful ulcer (VAS 8/10), necrosis, anaemia, elevated CRP | Venous ulcer | Metastatic carcinoma of unknown primary | Palliative oncology consultation, symptom-oriented wound care | Non-healing; palliative management |
| 6 | 65 / F | 14 months | Ulcer arising in long-standing scar (>30 years), moderate pain (VAS 5/10) | Scar-related ulcer | Squamous cell carcinoma (Marjolin ulcer) | Wide local excision, skin grafting | Complete healing; no recurrence at 12 months |
| 7 | 58 / M | 8 months | Worsening ulceration within known MF plaques, pain (VAS 6/10) | MF flare / infection | Mycosis fungoides progression | Systemic interferon-α, wound care | Slow healing with recurrent flares |
| 8 | 62 / F | 10 months | Persistent ulceration in MF context, eosinophilia, moderate pain (VAS 4–5/10) | MF flare | Mycosis fungoides with ulceration | Bexarotene, topical steroids, phototherapy | Gradual improvement |
